# Pseudo-outbreak of adenovirus in bronchoscopy suite

**DOI:** 10.1017/ice.2021.129

**Published:** 2021-04-08

**Authors:** Jessica L. Seidelman, Ibukunoluwa C. Akinboyo, Bonnie Taylor, Nancy G. Henshaw, Anfal Abdelgadir, Gregory C. Gray, Becky A. Smith, Sarah S. Lewis

**Affiliations:** 1 Division of Infectious Diseases and International Health, Department of Medicine, Duke University School of Medicine, Duke University, Durham, North Carolina, United States; 2 Duke Center for Antimicrobial Stewardship and Infection Prevention, Duke University Medical Center, Durham, North Carolina, United States; 3 Division of Infectious Diseases and International Health, Department of Pediatrics, Duke University School of Medicine, Duke University, Durham, North Carolina, United States; 4 Department of Pathology, Duke University School of Medicine, Duke University, Durham, North Carolina, United States; 5 Duke Global Health Institute, Duke University, Durham, North Carolina, United States; 6 Global Health Research Center, Duke Kunshan University, Kunshan, China

Inadequate high-level disinfection (HLD) and sterilization of endoscopes can result in contaminated bronchoalveolar lavage (BAL) fluids leading to transmission of pathogens that colonize or infect susceptible patients. To date, only 1 prior publication has described an adenovirus pseudo-outbreak associated with bronchoscopes.^1^ In January 2020, clinicians at an academic hospital in the southeastern United States noted a cluster of adenovirus polymerase chain reaction (PCR)–positive BAL samples, which prompted our outbreak investigation and subsequent mitigation.

## Methods

Initially, 5 inpatients at our 957-bed tertiary-care hospital were observed to have adenovirus-positive BAL specimens over a short period. The infection prevention team subsequently launched an investigation of microbiology results from October 1, 2019, to January 24, 2020, to determine the baseline prevalence of adenovirus-positive BAL results by clinical location and identify additional cases involved in the observed cluster. An epidemiologic investigation was conducted. Medical charts were reviewed to determine symptom status at the time of positive BAL. Procedure logs were reviewed to identify scopes in common between patients and to identify additional patients exposed to implicated scopes. Direct observations were made of HLD practices and logs, endoscope storage, and general cleanliness of the bronchoscopy reprocessing area and clinic environment. A single sham BAL sample was collected by drawing sterile saline through the suction channel to mimic the collection of clinical specimens from each scope. These samples were epidemiologically linked to the positive cases.

Initial diagnostics were performed with commercial DNA extraction, NucliSENS easyMag, (bioMerieux, Durham, NC), following the manufacturer’s instructions. The clinical and infection control specimens were examined with eSensor Respiratory Viral Panel (RVP; GenMark Diagnostics, Carlsbad, CA) platform using the company’s proprietary competitive DNA hybridization and electrochemical detection multiplex assay and XT-8 instrumentation. The Duke One Health Laboratory further studied the adenovirus-positive clinical specimens from 9 of the 10 patients by screening them with conventional PCR and subsequent Sanger sequencing, along with cell culture inoculations in A549 cells (2 passages). Multiple attempts were made to type the adenovirus DNA from original clinical specimens and culture using the procedures described in McCarthy et al^2^ and Zhang et al.^3^ No cultures exhibited had evidence of adenovirus propagation.

**Fig. 1.**
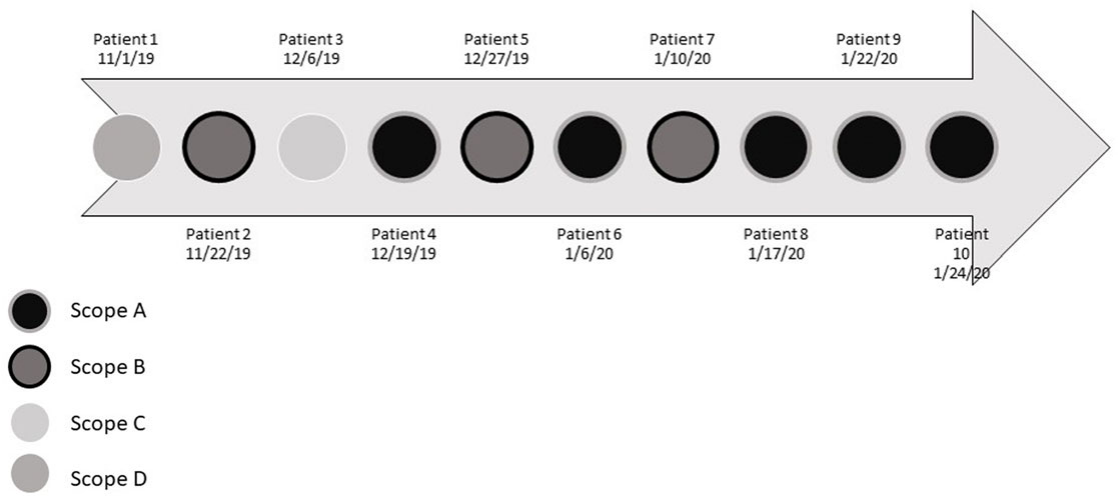
Timeline of 10 adenovirus-positive patients and affiliated scopes.

## Results

All inpatient bronchoscopies were performed in a single bronchoscopy suite. In total, 10 inpatients had positive adenovirus PCR results by multiplex PCR during the investigation period (Figure 1). Of 10 patients, 8 had bronchoscopies with 1 of 2 bronchoscopes (scope A or scope B) of the fleet of 8 bronchoscopes in this suite. The patient with the earliest adenovirus-positive BAL specimen had evidence of clinical disease, and the subsequent 7 patients were asymptomatic. Of 11 total patients who had bronchoscopy with scope A and had adenovirus testing performed during this period, 6 (55%) had molecular evidence of adenovirus infection. Of 24 total patients who had bronchoscopy with scope B and had adenovirus testing performed during this period, and 4 (17%) were positive.

Sham BAL specimens from both bronchoscopes tested negative for adenovirus by PCR. Of 10 patient BAL samples, 9 were validated with conventional PCR as having molecular evidence for adenovirus. Of these samples, 3 yielded sequence data and were closely related to human mastadenovirus C, and all 3 specimens came from scope A.

Our in-depth review of reprocessing, endoscope handling and storage, and general cleanliness of the bronchoscopy reprocessing area and clinic environment did not yield any deficiencies. Bronchoscopes A and B were returned to the manufacturer for evaluation. On inspection, scope A failed both wet and dry leak tests and had several physical defects. Scope B passed both wet and dry leak tests and had minimal physical issues found on inspection. This report led to an internal investigation of leak testing in the bronchoscopy suite, which did not find any deficiencies at the time the review was conducted. After removal of both bronchoscopes from service, no additional positive adenovirus samples from the bronchoscopy unit were observed for the following 9 months.

## Discussion

Previously, very few pseudo-outbreaks of adenovirus have been linked to bronchoscopes. Although we obtained a limited number of sham BALs and were unable to identify adenovirus from the damaged scopes we believe that the epidemiology—particularly the fact that the outbreak ceased once the implicated scopes were removed from service—supports the premise that this cluster was a pseudo-outbreak related to 1 or more contaminated devices. Specifically, we hypothesize that our index patient with clinical disease and subsequent positive cultures in the setting of the internal damage to the bronchoscope found on the manufacturer’s investigation served as a nidus for endoscope contamination, rendered our standard HLD procedures ineffective, and resulted in secondary contamination of the clinical samples.

We acknowledge that our investigation was limited by the inability to isolate adenovirus from the bronchoscope and confirm a definitive origin of the pseudo-outbreak. In addition, we could not confirm that the cases all involved the same adenovirus strain, but 3 of 6 samples from scope A appear to be closely related.

Notably, we were unable to detect the internal damage of scope A despite adherence to our routine leak testing and reprocessing protocols. However, several reports document outbreaks related to endoscopes despite adherence to meticulous stepwise reprocessing.^4,5^ Several adjunct interventions have been suggested to verify reprocessing efficacy, including visual inspection with borescopes.^6,7^ In fact, a growing body of literature indicates that borescope inspection of the internal endoscope components may improve reprocessing success.^8^ However, whether regular visual inspection of endoscopes with borescopes to detect internal luminal damage is an effective strategy to reduce endoscope contamination leading to pseudo-outbreaks or outbreaks is unknown and is a potential area for future research.

Bronchoscopy-related pseudo-outbreaks occur despite standardized procedures for HLD.^1,9^ New technology that is high-quality disposable or able to undergo sterilization is needed. Until such technology exists, bronchoscopy clinics, particularly those with a high volume of immunocompromised patients, should prospectively review BAL cultures to identify unusual pathogen trends. These trends may be a sign of damaged equipment or failures in HLD that would otherwise go undetected.
